# Direct Graft Copolymerization of Cellulose Acetate Membrane with Bio-Based Itaconic Acid for Pollutant Removal from Wastewater

**DOI:** 10.3390/membranes16080276

**Published:** 2026-08-18

**Authors:** Abir S. Abdel-Naby, Salsabeel S. Abo-Ghonaim, Salha N. Alharthi, Hagar H. Alhaddad, Nuhu Dalhat Mu’azu

**Affiliations:** 1 Department of Pharmaceutical Chemistry, College of Pharmacy, Imam Abdulrahman Bin Faisal University, P.O. Box 1982, Dammam 34221, Saudi Arabia; 2200006263@iau.edu.sa; 2 Department of Chemistry, College of Science, Imam Abdulrahman Bin Faisal University, P.O. Box 1982, Dammam 31441, Saudi Arabia; 2230500254@iau.edu.sa (S.S.A.-G.); snalharthi@iau.edu.sa (S.N.A.); 3 Environmental Engineering Department, College of Engineering, Imam Abdulrahman Bin Faisal University, P.O. Box 1982, Dammam 31451, Saudi Arabia; nmdalhat@iau.edu.sa

**Keywords:** cellulose acetate, itaconic acid, phase inversion, graft copolymerization, water purification

## Abstract

Cellulose acetate (CA) is a promising bio-derived membrane material for water treatment; however, its limited availability of active functional sites can restrict its affinity toward dissolved pollutants. In this study, a cellulose acetate membrane was fabricated by phase inversion and subsequently functionalized through novel direct graft copolymerization with bio-based itaconic acid (IA) using potassium persulfate (KPS) as an initiator in an aqueous medium. The grafting approach introduced carboxylic functional groups into the CA matrix, providing additional active sites for pollutant removal. The successful grafting was confirmed by UV–Vis and 1H NMR spectroscopy, while XRD indicated changes in the structural organization of the polymer matrix. SEM/EDS characterization further revealed morphological changes associated with grafting, and cross-sectional SEM showed the development of finger-like, continuous pore channels within the modified membrane. The effects of reaction time, IA concentration, and KPS concentration on the grafting percentage were systematically evaluated, with grafting increasing up to an optimum range before declining at excessive monomer or initiator concentrations. Thermal analysis demonstrated improved stability after grafting, with the 6.6% grafted CA-g-IA membrane exhibiting an initial decomposition temperature of 351 °C and a reduced weight loss of 85% at 500 °C, compared with 344 °C and 91%, respectively, for pristine CA. The 6.6% CA-g-IA membrane was subsequently evaluated for the removal of Cu(II) and methylene blue (MB) from aqueous solutions. Cu(II) uptake was strongly influenced by contact time, solution pH, initial concentration, and grafting percentage, with the highest performance observed around pH 6 and 240 min contact time. The membrane also maintained its Cu(II)-binding performance over four regeneration cycles following HNO_3_ treatment. Overall, direct IA grafting provides a simple bio-based functionalization strategy for enhancing the pollutant-binding functionality of cellulose acetate membranes, demonstrating potential for the removal of metal ions and cationic dyes from contaminated water.

## 1. Introduction

Biopolymers are attracting growing attention as sustainable alternatives to petroleum-based polymers due to their eco-friendliness, renewability, and biodegradability [1,2]. Among biopolymers, natural polysaccharides such as cellulose, starch, chitin, and chitosan are of particular interest for pharmaceutical, biomedical, packaging, energy, and wastewater treatment applications [3,4,5,6,7,8,9,10]. Cellulose stands out as the most abundant biopolymer, and it is valued for its thermal stability, non-toxicity, chemical modifiability through its hydroxyl groups, enabling the formation of useful derivatives such as cellulose acetate and carboxymethyl cellulose [11,12,13,14,15,16,17,18,19,20,21,22,23]. Different techniques have been explored to address the poor solubility of cellulose which arises from strong hydrogen-bonding within its structure including chemical modification, graft copolymerization, polymer composites, and polymer blending [24,25,26]. Amongst the chemical modification techniques, nitration, esterification, oxidation, and acetylation are effective methods for incorporating functional groups that enhance solubility, improve membrane performance and promote efficient pollutants removal from water [27,28,29,30,31,32,33,34,35,36]. Graft copolymerization converts cellulose into a multifunctional material by incorporating polymers leading to materials with superior thermal stability and improved selectivity toward specific contaminants [37,38,39,40,41,42,43,44,45,46,47,48,49,50]. In addition, cellulose-based composites reinforced with hydroxyapatite (HAp), graphene oxide (GO), carbon nanotubes (CNTs), and titanium dioxide (TiO2) have also shown enhanced mechanical properties, and strong affinity for heavy metals. Likewise, blending cellulose derivatives with other polymers has been employed to inherit desirable properties such adsorption capacity (GO, HAp), conductivity (CNTs), and photocatalytic activity (TiO_2_) without altering its native chemical structure [51,52,53,54,55,56]. The integration of these techniques has broadened the applications of cellulose-based materials in membrane systems and environmental remediation including oil–water separation and desalination processes. Cellulose acetate (CA)-based membranes have become a promising material for wastewater applications due to their biodegradable nature, hydrophilic properties, and film fabrication capability [28]. The integration of additives such as TiO_2_ nanorods, metal–organic frameworks, ZrO_2_ nanoparticles, MXenes, and halloysite nanotubes can enhance desalination performance by improving slat rejection, and surface hydrophilicity [55,57,58,59,60,61,62,63]. For heavy metal removal, modified cellulose-based materials including TEMPO-oxidized nanocellulose, ZnO-modified CA membranes, Fe_3_O_4_-functionalized cellulose, TiO_2_-containing nanofibers, and hydroxyapatite composite have exhibited strong adsorption capacities toward heavy metals ions such as Cd(II), Cu(II), Pb(II), and Fe(III) from wastewater [21,43,46,64,65,66,67,68,69,70,71,72,73]. In reverse osmosis systems, cellulose-derived nanomaterials have been incorporated into polyamide thin-film composite membranes to enhance desalination capability, chlorine resistance, and mechanical strength [74,75]. Likewise, PVC/CA and TiO_2_-modified CA membranes have shown high salt rejection rates up to 99% [56,76,77]. Among emerging sustainable additives, itaconic acid (IA) represents a promising alternative to acrylic acid in the development of hydrogels and advanced polymeric adsorbents for removal of heavy metal ions, including Cu(II), Cd(II), Zn(II), and Pb(II) [78,79,80,81,82,83,84].

Although cellulose acetate has been modified extensively with a variety of nanomaterials for water treatment applications, the integration of itaconic acid remains relatively underexplored. Due to its bio-based nature and dual carboxylic acid functionality, itaconic acid offers significant potential for improving membrane hydrophilicity and adsorption performance of cellulose acetate membranes for wastewater remediation technologies.

Unlike previous graft copolymerization onto CA solution, where the grafted product should be precipitated, washed with the monomer solvent through the Soxhlet system and then dried, followed by dissolving the grafted cellulose acetate into DMF to be fabricated into membrane [61], the main novelty of this work is the direct graft copolymerization of bio-based itaconic acid (IA) onto a cellulose acetate (CA) membrane using KPS initiation after membrane fabrication, creating various removal active sites through the membrane. This will reduce the use of solvents and non-solvents and enhance the product yield.

## 2. Materials and Methods

### 2.1. Instruments

The characterization of compounds via nuclear magnetic resonance spectroscopy utilized a 400 MHz NMR device (Bruker Corporation, Bremen, Germany), employing DMSO-d6 as a solvent for sample dissolution. The SHIMADZU UV-1601, UV/visible spectrophotometer, Japan. operating in the wavelength range of 200–400 nm, was used to determine the concentration of the adsorbed material. Scanning electron microscopy images were taken with a TESCAN (Brno, Czech Republic) VEGA3 scanning electron microscope, with a secondary electron (SE) detector, an energy-dispersive spectrometer detector, and electron backscatter diffraction (EBSD). The thermal decomposition of the samples was studied using a simultaneous thermogravimetric and differential thermal analyzer (DTG60H, SHIMADZU corporation, Kyoto, Japan) under a N_2_ atmosphere at a rate of 5 ◦C/min, between 25 and 500 °C. To obtain elemental information, energy-dispersive X-ray fluorescence spectrometry (EDX8000, SHIMADZU corporation, Kyoto, Japan) was employed. To investigate the mechanical properties of membranes, a tensile testing machine (DCP-KZ300, Sichuan, China) was used to test their tensile strength and elongation-at-break. The speed of the crosshead was 20 mm/min. The fabricated membrane was snipped into a rectangle shape with a width of 15 mm and a total length of 100 mm. The sample of the membrane was tested at room temperature. The hydrophilicity of the fabricated membrane surface was measured as a function of the contact angle measurements using Rame Hart Goniometer, Model 290, France. A distilled water drop (2 μL) was added to the surface of the membrane (3 cm × 2 cm) using a micro syringe. The contact angle was measured by adding the drop of water, at five different positions, within 20 s, on the membrane surface. All the chemicals and reagents employed in these experiments were directly used as received without further purification.

### 2.2. Chemicals

Cellulose acetate (CA; average Mw ≈ 20,000, ≥75% degree of deacetylation) was supplied by Santa Cruz Biotechnology (SCBT), USA. Potassium persulfate (KPS) and itaconic acid (IA) were obtained from Winlab Limited, India and Riedel-de-Haën, Germany respectively, while cupric sulfate was supplied by BDH Chemicals Ltd., Poole, UK. Nitric acid, methylene blue (MB), sodium hydroxide, and ammonium hydroxide were purchased from Sigma-Aldrich, USA. All aqueous solutions were prepared using double-distilled water.

### 2.3. Procedures

#### 2.3.1. Fabrication of the Membrane

The phase inversion technique was employed for the membrane fabrication. A 13 wt.% cellulose acetate solution in DMF was cast onto a glass plate using an automatic film applicator equipped with a 200 μm casting knife at 25 °C and 4 rpm. The cast film was subsequently immersed in distilled water to induce phase inversion, after which the formed membrane was detached and soaked in distilled water overnight.

#### 2.3.2. Synthesis of Cellulose Acetate Graft Itaconic Acid Copolymers

##### Pre-Activation of CA Membrane

The pre-activation of the CA membrane surface was carried out by treating it with 0.014 wt.% NaOH for 2 min to induce partial deacetylation [85], after which, the membrane was rinsed thoroughly with DI water to facilitate the subsequent grafting process.

The dried grafted membrane was weighed, and the grafting percentage (G%) was calculated using Equation (1) [86].

(1) $$G\%=\frac{W-W_{0}}{W_{0}}\times 100$$

where:

*W*_0_ represents the initial weight of the polymer before grafting, while *W* corresponds to the weight of the grafted copolymer.

##### Grafting of CA Membrane

In a preheated water bath (60 °C), (0.2 ± 0.003 g) with a definite area of pre-activated (CA) membrane was placed in DI water in a double-necked round-bottom flask, in which a reflux condenser was inserted to prevent water loss. After applying N_2_ gas for 10 min, a certain concentration (0.0012, 0.0016, 0.0019, 0.0023 M) of potassium persulfate (K_2_S_2_O_8_) solution was placed into the flask. After 30 min, a certain concentration (0.076, 0.134, 0.173, 0.23, 0.32 M) of itaconic acid solution was poured into the flask. The graft copolymerization was allowed to react, while maintaining reaction conditions (60 °C, N_2_ atmosphere, total 200 mL of DI water), for various reaction times.

The membrane pieces were washed thoroughly with DI water to remove unreacted itaconic acid.

After filtering the membranes using vacuum filtration, the membranes were allowed to dry at room temperature.

##### Membrane Performance

To investigate the water permeability of the membrane, the water flux was calculated using Equation (2) [61].

(2) $$J=\frac{\Delta V}{A\Delta t}$$

where Δ*V* (L) is the permeate volume; *t* (h) is the filtration time interval; and *A* (m^2^) is the area of membrane.

#### 2.3.3. Processes for Removal of the Metal Ions 

##### Preparation of the Stock Solution of Metal Ions

A standard Cu(II) stock solution (200 ppm) was diluted using double-distilled water to prepare the required concentrations.

##### Metal Removal Process

A definite weight of membrane G% (0%, 6.6%, 13%, 16%) was placed in 20 milliliters of a buffer solution with a specific pH value (5, 6, and 7.5) containing an aqueous solution of Cu(II) concentration (50, 100, 200 ppm). The mixture was then shaken in a shaker at 155 rpm at room temperature for various durations (30, 120, 240, 360 min).

After shaking, the mixture was separated by filtration. The amount of metal cations removed was determined using Equation (3) [86].

(3) $$q=\frac{\left(C_{0}-C_{f}\right)}{m}\times v$$

where

(*C*_0_ − *C_f_*): the difference between the initial metal concentration and the final metal concentration (after the removal process) (ppm), respectively.

*v:* the volume of the aqueous solution (l).

*m*: mass of the membrane (g).

*q*: the removal capacity (mg/g).

*q* was determined by the difference in CuSO_4_ concentration before and after the adsorption process, measured using a calibration curve of Cu(II) absorbance. Prior to UV/Vis analysis, ammonia was added to form the [Cu(NH_3_)_4_]^+2^ complex, enhancing the detection sensitivity, while the residual metal content in the polymeric matrix was qualitatively assessed using EDS.

#### 2.3.4. Reusability of the Grafted Membranes

To evaluate the regeneration performance of cellulose acetate grafted itaconic acid (CA-g-IA) membrane, 0.1 M of nitric acid (68–70%) was added to leach out the Cu (II) from the membrane’s matrix.

During the leaching process, 0.02 g of membrane was immersed in 10 mL of acid solution and shaken in a thermostatic shaker at 150 rpm for 2 h at a slightly elevated temperature [61,86]. After treatment, the membrane was removed, thoroughly rinsed with DI water, and dried at room temperature. Post-regeneration, membrane samples were analyzed by EDS to confirm the removal of metal cations.

#### 2.3.5. Dye Removal Procedure

A definite weight of (CA-g-IA) membrane was shaken with 20 mL of dye solution (40 ppm) for 2 h at the dye pH solution.

The removal capacity of the grafted membranes was calculated by Equation (2) [81].

Where *(C*_0_ *− C_f_):* the difference between the initial dye concentration and the final dye concentration (after the removal process) (ppm), respectively.

The concentration of the solution was determined by a calibration curve, using a UV/vis spectrophotometer.

The membrane after the removal process was examined using EDS.

## 3. Results and Discussion

The suitability of polymeric membranes for water treatment depends on key properties such as biodegradability, thermal stability, and the availability of functional groups that can interact with target pollutants.

Cellulose acetate (CA) is a biodegradable, semi-crystalline polymer with good film-forming characteristics, making it suitable for membrane fabrication through the phase-inversion technique. However, pristine CA exhibits limited thermal stability at elevated temperatures, with substantial weight loss occurring by 500 °C. In addition, its polymeric structure provides relatively few functional sites for effective pollutant binding.

Therefore, chemical modification of CA is required to enhance its functionality while retaining its favorable membrane-forming properties. In this study, graft copolymerization was employed to introduce additional functional groups into the CA matrix and improve its potential for pollutant removal.

### 3.1. Graft Copolymerization of CA Membrane with the IA Monomer

The structure of itaconic acid (IA) exhibits organic moieties including two carboxylic (-COOH) groups and a double bond. The carboxylic groups exhibit the ability to attract cations through the formation of ionic or coordination bonds [87,88]. Also, the presence of a double bond would enhance the graft copolymerization. Thus, the modification of CA by IA is expected to show optimized properties for water treatment applications.

### 3.2. Characterization of CA-g-IA

#### 3.2.1. Spectroscopic Analysis

**Figure 1:**
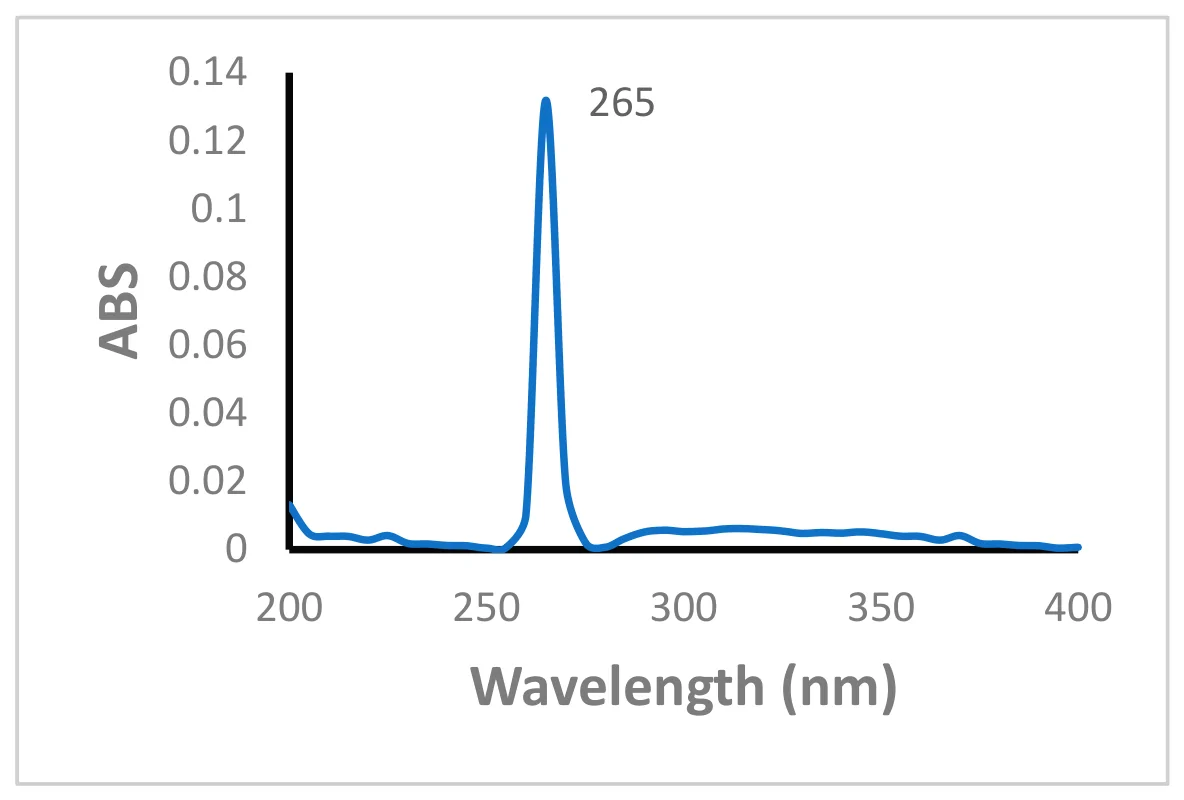
UV/vis spectrum of CA-g-IA (2.2 G%) in DMF (1 mg/1 mL).

The modified polymer CA-g-IA was characterized using spectroscopic analysis. The UV/vis spectrum of CA-g-IA exhibited a distinct absorption peak at around λ = 265 nm related to the itaconic acid moieties (Figure 1), as compared to CA, which does not absorb in the UV-vis range.

**Figure 2:**
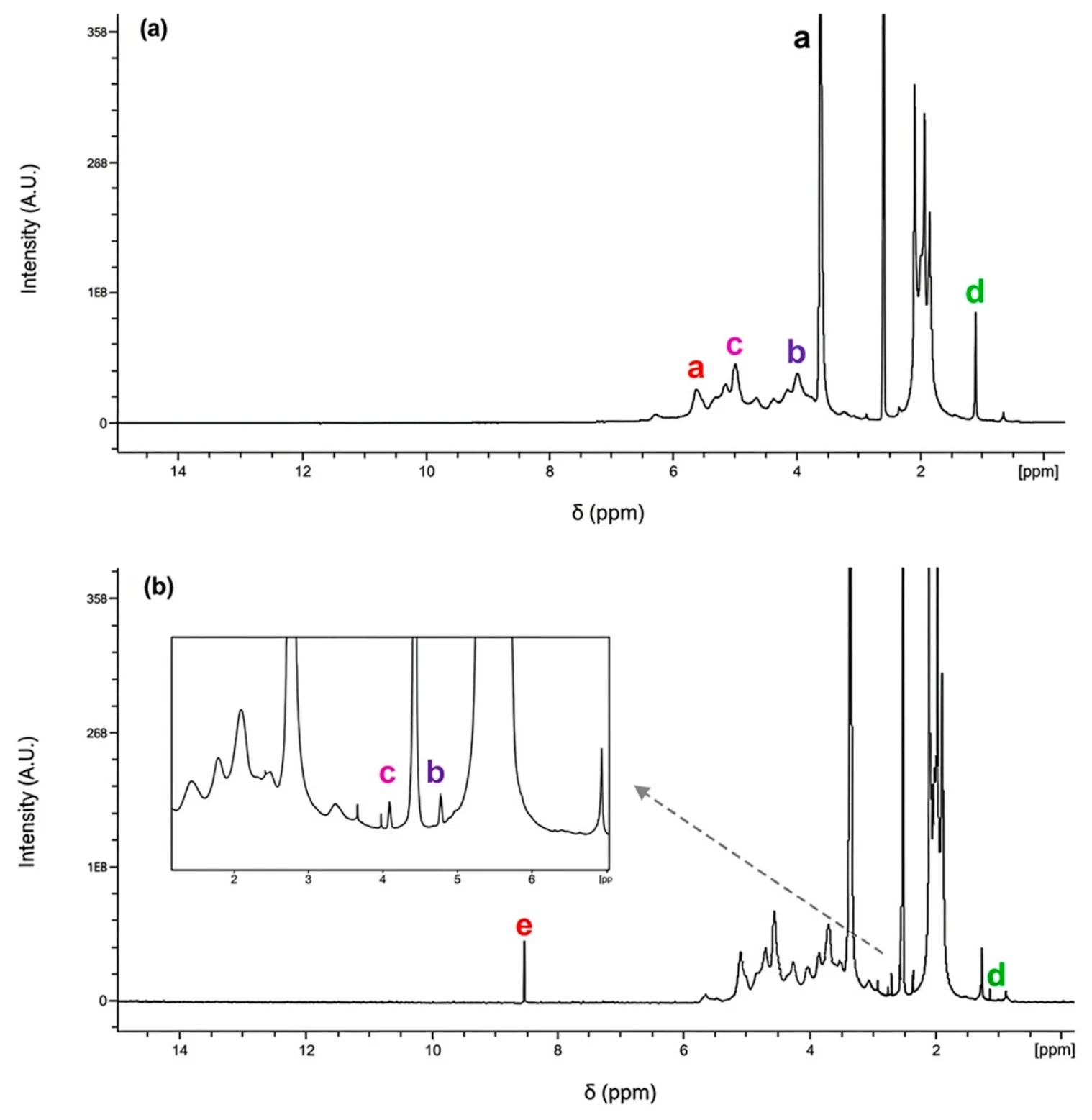
^1^H NMR of CA-g-IA (**b**) as compared to that of CA (**a**).

Figure 2b showed the ^1^H NMR spectrum of (CA-g-IA) as compared to that of CA (Figure 2a). The (CA-g-IA) spectrum showed that the carboxylic proton was present at 8.6 ppm, which confirmed that the carboxylic group remained untouched during the modification reaction.

The upfield shift of the carboxylic proton was attributed to its isolation between the cellulose acetate main chains, which significantly reduces hydrogen bonding. Additionally, the appearance of the carboxylic proton peak was considered direct evidence of graft copolymerization. The ^1^H NMR spectrum of the IA monomer does not show this peak due to extensive inter- and intramolecular hydrogen bonding between IA molecules, as well as the probability of interaction with deuterated solvent through the formation of hydrogen bonding, too [89].

The copolymerization of IA monomers was also confirmed by the presence of saturated protons (Hb and Hc) at 2.4 and 2.7 ppm, respectively (Table 1).

**Scheme 1:**
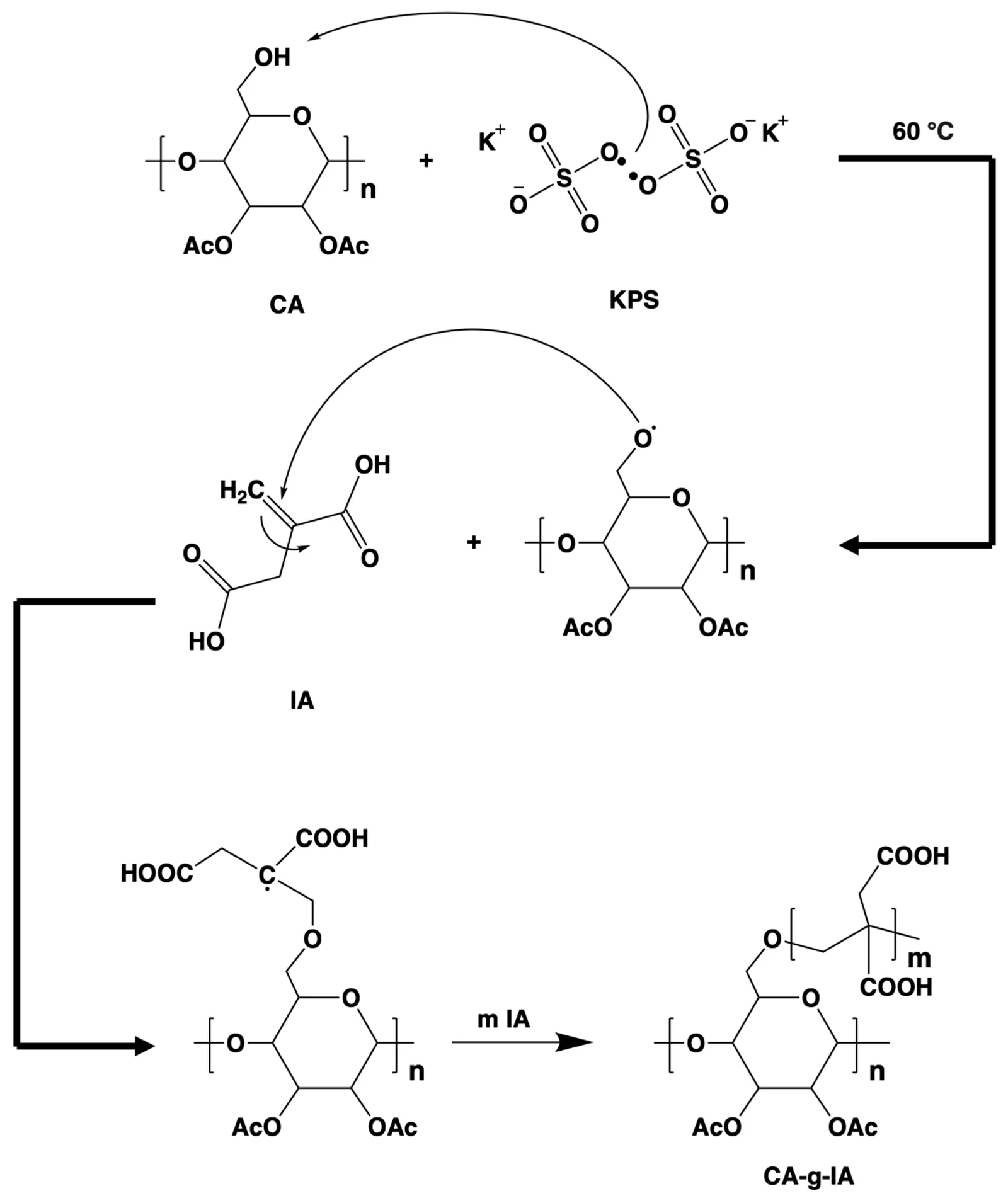
Graft copolymerization mechanism of IA onto CA membrane.

A suggested mechanism for the graft copolymerization is illustrated in Scheme 1.

The reaction occurred between the hydroxyl groups of CA and the double bond of IA in the presence of the persulfate initiator. The graft copolymerization would build branches of IA moieties onto the polymer main chain, thus affording a considerable number of carboxylic groups through the CA matrix, which would enhance the removal of target pollutants, such as metal cations or dyes, from aqueous solutions.

#### 3.2.2. XRD of CA-g-IA

**Figure 3:**
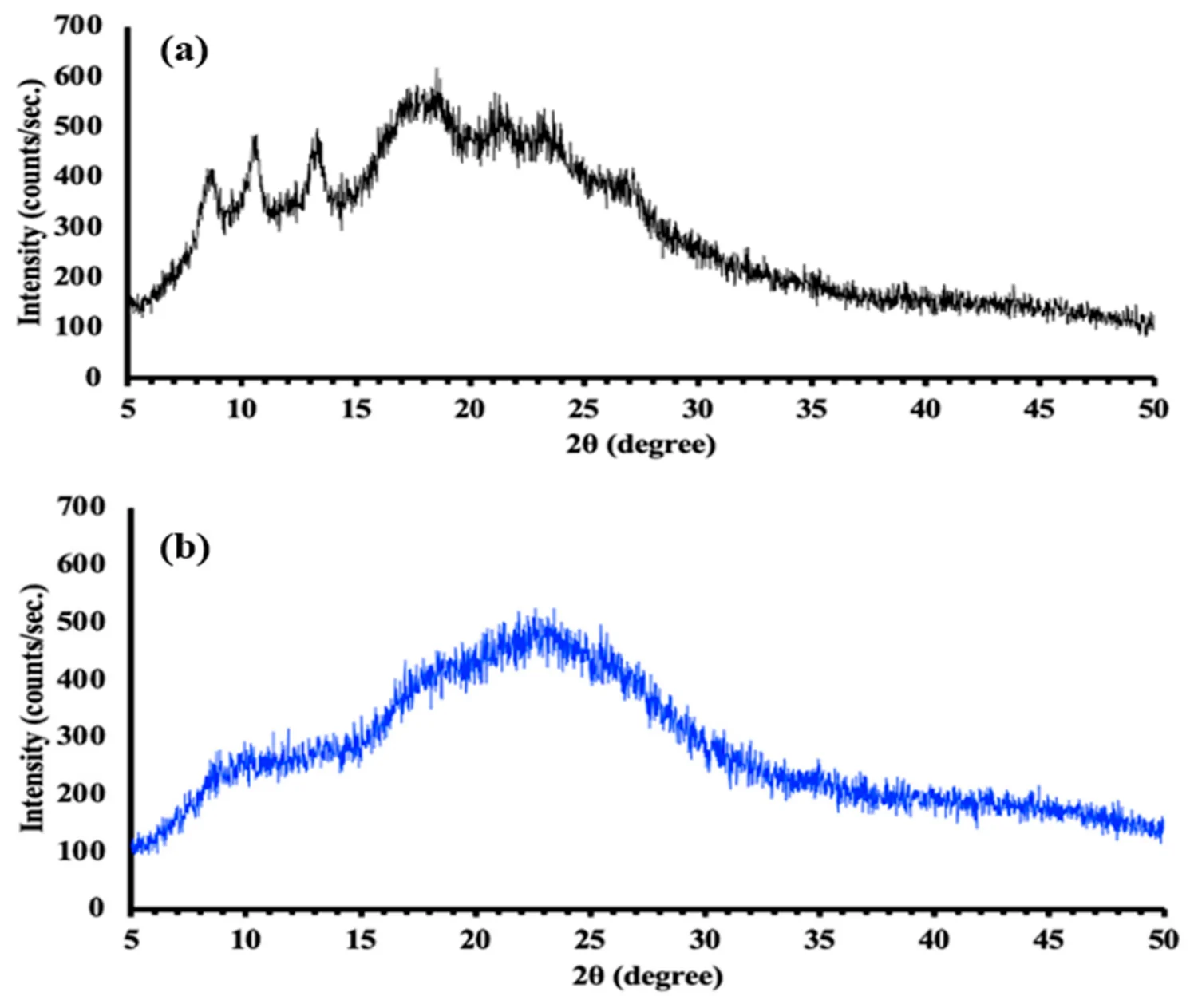
XRD patterns of (**b**) CA-g-IA as compared to that of the pristine CA (**a**).

The CA-g-IA membrane was further characterized by X-ray diffraction (XRD) analysis to evaluate structural changes in the polymeric matrix following graft copolymerization. Figure 3a shows sharp peaks at 2Ɵ < 15, referring to the crystalline regions of the polymeric matrix, while the broad bands at 2Ɵ > 17 correspond to the amorphous regions [76]. The modified cellulose acetate (Figure 3b) shows only broad peaks, which indicates a reduction of the crystallinity of the polymeric matrix, which is attributed to the substitution of the hydroxyl groups by the branches of IA moieties [61].

#### 3.2.3. Morphological Characterization of CA-g-IA

**Figure 4:**
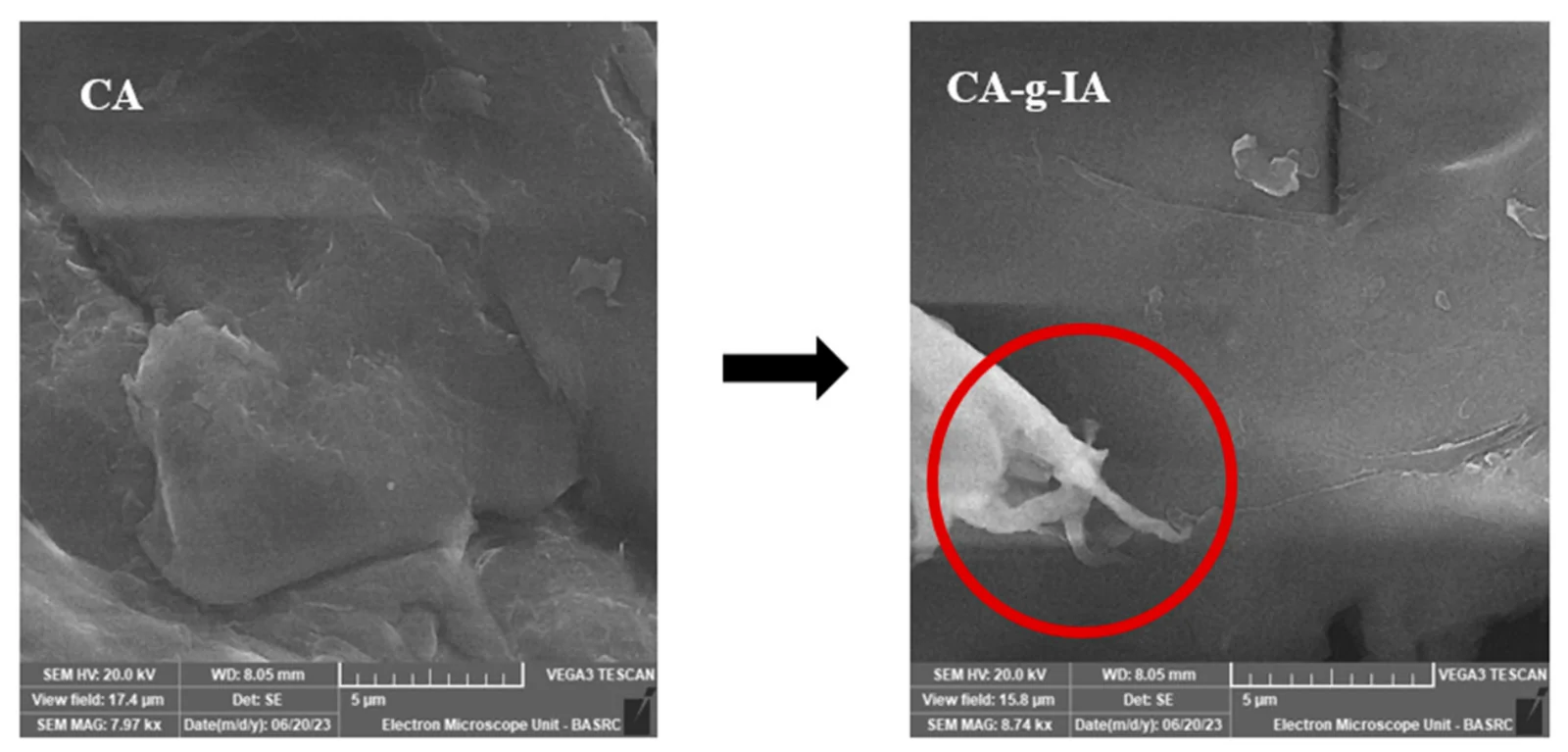
SEM images of the surface morphology of CA-g-IA membrane, as compared to that of un-grafted CA membrane. The circle refers to the graft parts.

An effective confirmation of the grafting process into the polymeric matrix of CA was provided by the SEM morphological characterization (Figure 4). SEM analysis of the CA-g-IA membrane revealed distinct grafted regions distributed over the cellulose acetate polymeric matrix, as highlighted in the micrograph.

### 3.3. Factors Affecting the Graft Copolymerization Percentage

To optimize the graft copolymerization process, the effects of key reaction parameters on the grafting percentage (G%, calculated according to Equation (1)) were systematically investigated and results presented below.

#### 3.3.1. Effect of Time

**Figure 5:**
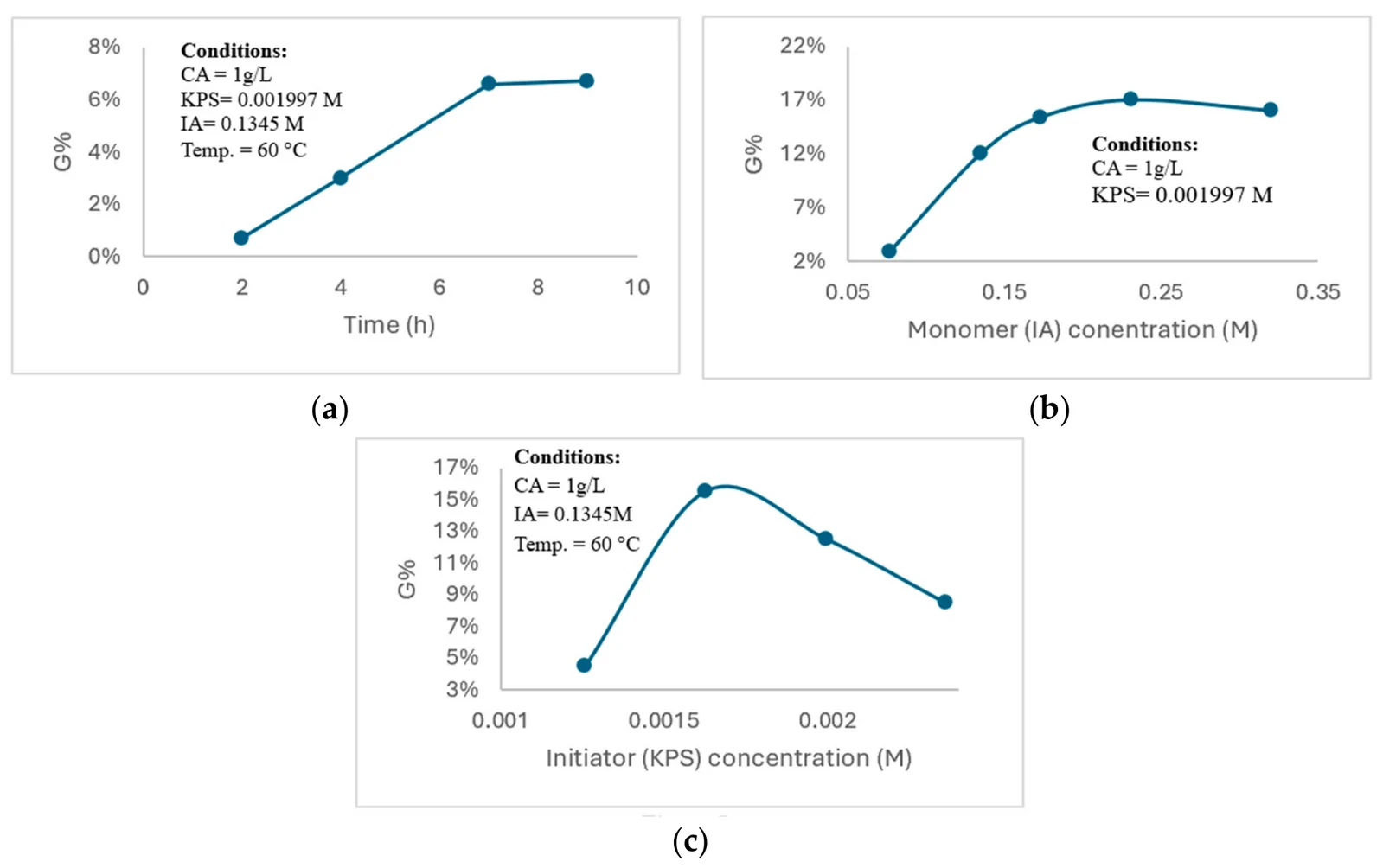
Factors affecting the graft copolymerization percentage membrane. (**a**) Effect of time (**b**) Effect of the monomer concentration (**c**) Effect of the initiator concentration.

The effect of reaction time on the grafting percentage of IA onto the CA backbone is presented in Figure 5a. The grafting percentage gradually increased with reaction time, reflecting the progressive growth of IA branches within the CA matrix. A plateau was reached after approximately 7 h, likely due to the gradual consumption of available monomer during the formation and growth of the grafted polymeric branches [90].

#### 3.3.2. Effect of Monomer Concentration

Figure 5b illustrates the effect of IA monomer concentration on the grafting percentage (G%). Increasing the monomer concentration enhanced G%, reaching a maximum at 0.23 M. Beyond this concentration, G% decreased, which may be attributed to the increased tendency of IA to undergo homopolymerization rather than grafting onto the CA backbone [91].

#### 3.3.3. Effect of Initiator Concentration

As shown in Figure 5c, the grafting percentage was strongly dependent on the KPS concentration. G% increased progressively as the initiator concentration increased, attaining its highest value at 0.0016 M KPS. Further addition of KPS resulted in a marked decline in grafting, possibly because the higher radical concentration promoted radical termination through recombination, thereby limiting further growth of the grafted chains [92,93].

Based on the preceding results, the selected conditions for graft copolymerization were 60 °C, 0.23 M IA monomer, and 0.001997 M KPS initiator. Before selecting the most suitable (optimal) grafting percentage for water treatment applications, the thermal stability of the modified membranes was evaluated using thermogravimetric analysis (TGA).

### 3.4. Thermal Properties of the Graft Membranes

**Figure 6:**
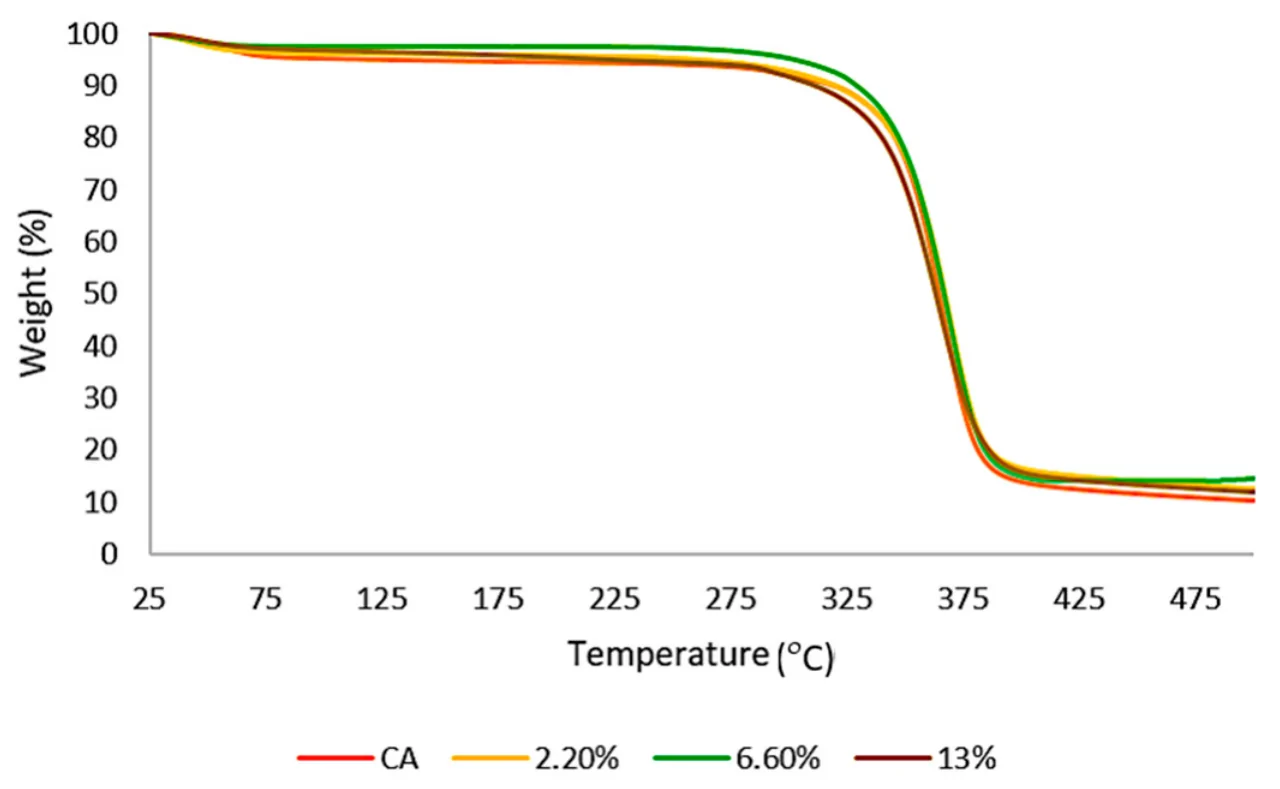
TGA of various graft percentages of CA-g-IA membranes, as compared to the ungrafted CA membranes.

The TGA profiles demonstrated improved thermal stability of the grafted membranes, as evidenced by the increase in the initial decomposition temperature (T_0_) (Table 2) and the lower percentage of weight loss at 500 °C (Figure 6).

Increasing the grafting percentage lead to enhancement of the thermal stability of the modified membranes at elevated temperatures. The CA-g-IA membrane with 6.6% grafting exhibited a weight loss of 85% at 500 °C, compared with 91% for pristine CA, indicating improved thermal resistance (Table 2). However, increasing the grafting percentage to 13% resulted in a slightly higher weight loss of 89%. This behavior may be associated with intermolecular interactions or crosslinking among the grafted polymer chains, together with disruption of the native crystalline regions of CA at higher grafting levels. These structural changes could promote earlier thermal degradation and consequently reduce the thermal stability of the highly grafted membrane [94,95].

**Figure 7:**
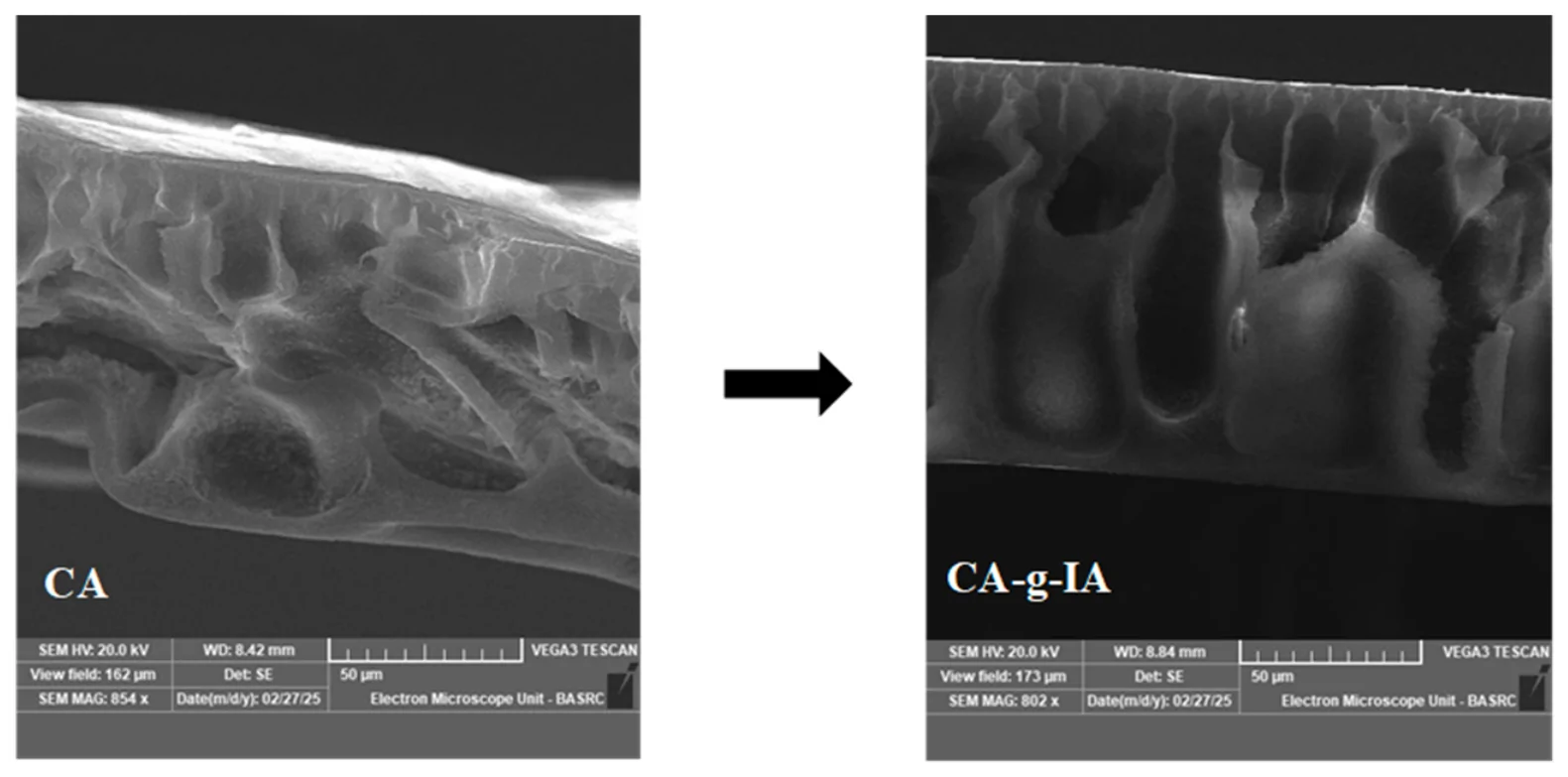
Cross-section of CA-g-IA (%G = 6.6) membrane as compared to that of CA membrane.

Prior to evaluating the grafted membranes for water treatment applications, their cross-sectional morphology was examined to assess the internal pore structure and potential pathways for water transport. As shown in Figure 7, the pristine CA membrane exhibited predominantly sponge-like pores with some channel-like structures. In contrast, the CA-g-IA membrane displayed more pronounced finger-like and continuous pore channels extending through the polymeric matrix. This interconnected morphology provides favorable pathways for water transport and supports the potential application of the grafted membrane in water treatment.

### 3.5. Mechanical Properties of the Grafted Membranes

CA is known to exhibit good mechanical properties [61]. The graft copolymerization of the polymer showed branches built onto its main chains. Thus, prior to the application of the graft copolymer in water treatment, their mechanical properties should be investigated. Table 3 lists the data of the tensile strength and elongation at break of various percentages of the CA-g-IA membranes. The results confirmed the suitability of CA-g-IA as a water purification membrane, since it maintained the good mechanical properties of CA.

### 3.6. Membrane Performance

To investigate the membrane performance, the water contact angles and water flux were investigated.

#### Contact Angle Measurement

The water contact angle measurement of the pristine CA membrane demonstrated a moderate surface wettability (highest contact angle value). The graft copolymerization with itaconic acid monomer, which exhibits two polar groups, enhanced the surface hydrophilicity of the membrane (Table 4).

The graft copolymerization afforded the polymer with branches of repeating units, including series of carboxylic groups.

The carboxylic groups of the IA moieties increased the water flux, as they form hydrogen bonding with the water molecules (Table 4). Thus, the percentage of graft would directly affect the water flux. To measure the water flux (*J*), the following equation was used:

$$J=\frac{\Delta V}{A\Delta t}$$

where Δ*V* (L) is the permeate volume; *t* (h) is the filtration time interval; and *A* (m^2^) is the area of membrane.

### 3.7. Water Treatment Applications of CA-g-IA Membrane

The CA-g-IA (6.6 G%) was chosen for the water treatment applications.

#### 3.7.1. Removal of Cu (II) Cations from Polluted Water by CA-g-IA Membrane

The removal process occurred as described in the experimental section.

##### Characterization of the Removal of Cu (II)

**Figure 8:**
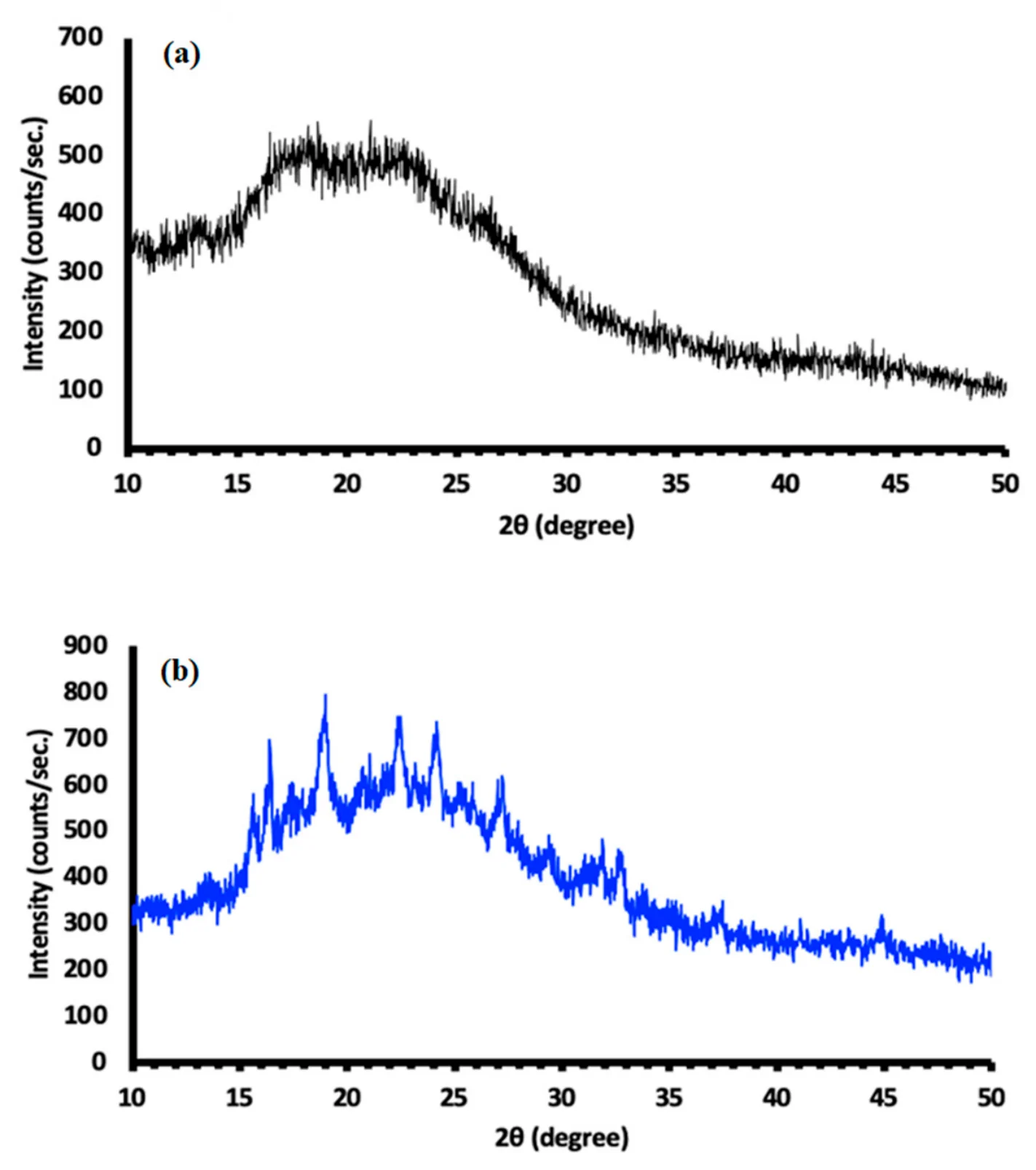
The XRD patterns of CA-g-IA membrane (5G = 6.6) before (**a**), and after (**b**) copper ion removal.

XRD analysis was used to verify Cu(II) uptake by the new CA-g-IA membrane. Following the removal process, the XRD pattern exhibited distinct sharp peaks associated with Cu(II) (Figure 8b) [96,97], whereas these peaks were absent in the membrane before Cu(II) exposure during the treatment process (Figure 8a).

**Figure 9:**
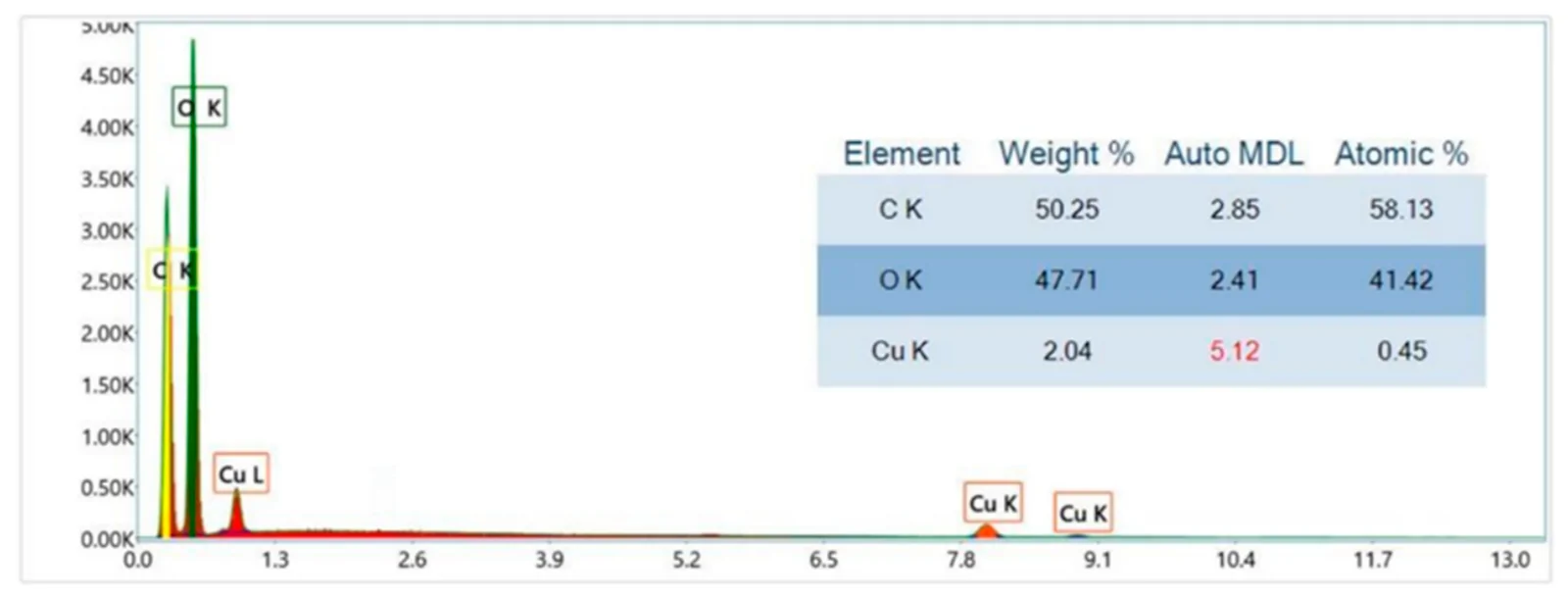
EDS of CA-g-IA membrane after the removal of copper ions.

EDS analysis further supported Cu(II) uptake by the grafted membrane. After the Cu removal process, distinct Cu-related peaks appeared in the EDS spectrum of CA-g-IA (Figure 9), confirming the presence of copper on the membrane.

To identify the conditions that favor enhanced Cu(II) removal by the CA-g-IA membrane, the effects of key operating parameters were systematically investigated.

##### Factors Affecting the Cu (II) Removal Process

To optimize Cu(II) removal by the CA-g-IA membrane, the influence of different operating parameters, which included contact time, pH, Cu(II) concentration and G%, were investigated. The membrane removal capacity was evaluated using the adsorption capacity (q) calculated according to Equation (2).

##### Contact Time

**Figure 10:**
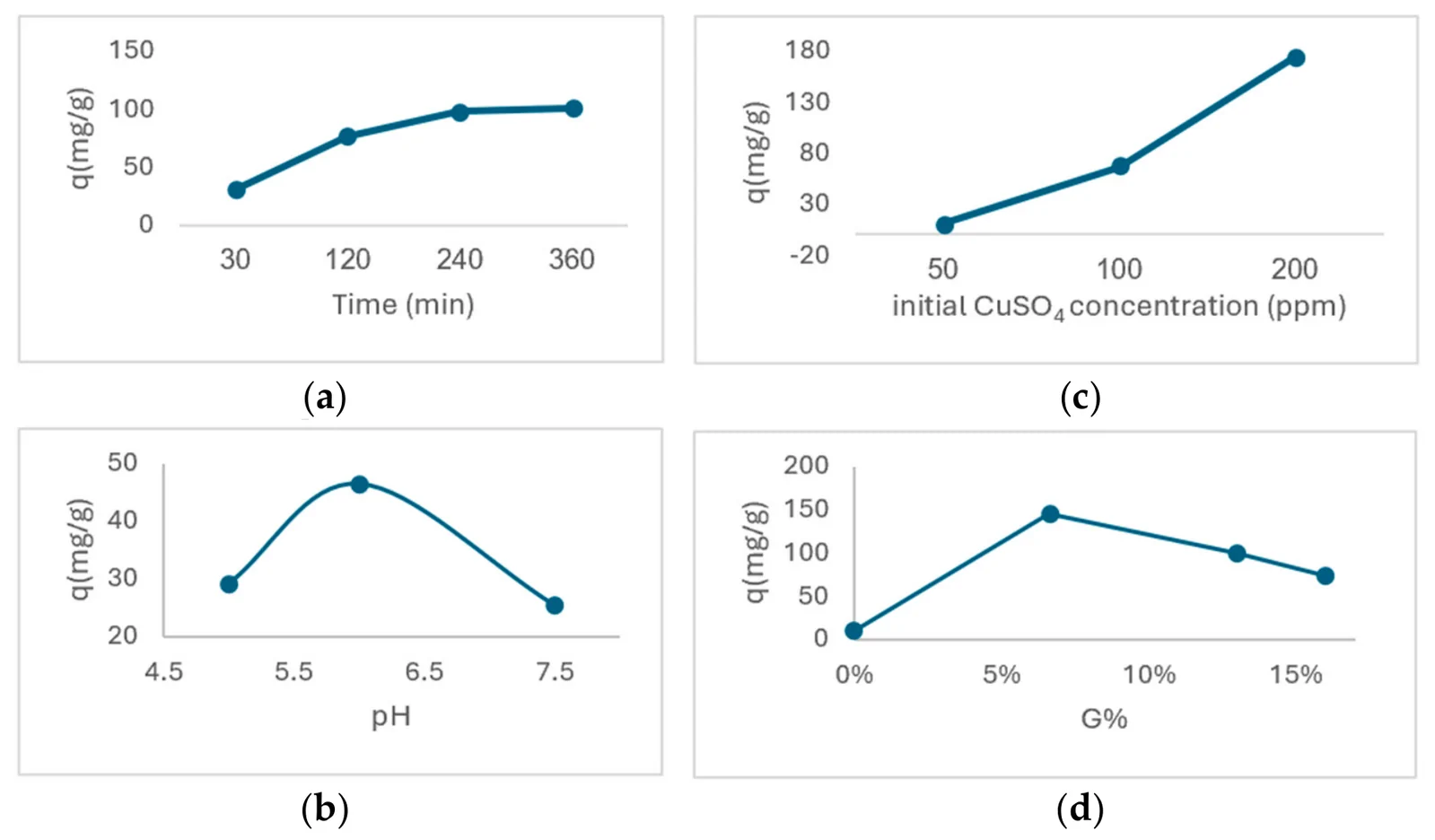
Factors affecting Cu (II) removal by CA-g-IA membrane. (**a**) Effect of time (**b**) Effect of pH (**c**) Effect of initial concentration of Cu (II) (**d**) Effect of graft percentage of CA membranes.

Figure 10a presents the influence of contact time on Cu(II) uptake by the CA-g-IA membrane. The adsorption capacity (q) increased progressively with contact time and approached its maximum at approximately 240 min, after which only a slight change was observed. This behavior indicates that most available binding sites had become occupied by Cu(II) ions, limiting further uptake with prolonged contact time [21,98].

##### Effect of pH

It has been well established that solution pH is a critical parameter governing Cu(II) adsorption [99]. As shown in Figure 10b, the adsorption capacity of the CA-g-IA (6.6%) membrane increased with pH and reached its maximum at pH 6. Under acidic conditions, excess H^+^ ions compete with Cu(II) for the available carboxylate binding sites, resulting in lower adsorption [100]. At alkaline pH, the observed decline in Cu(II) uptake was attributed to the increased formation of copper hydroxide species [21].

##### CuSO_4_ Solution Concentration

Figure 10c shows the effect of initial CuSO_4_ solution concentration on the removal process. The results revealed that the high concentration enhanced the removal capacity [98].

##### Effect of G%

The removal capacity of the membrane increased with the increase of the percentage of graft up to 6.6% (Figure 10d), which was attributed to the increase in the number of (-COOH) groups. A sharp decrease in the removal capacity was observed for a higher percentage of graft, which might be due to intra- or inter-hydrogen bonding of IA moieties inside the grafted branches [101].

#### 3.7.2. Application of CA-g-IA for Dye Removal from Aqueous Solution

**Figure 11:**
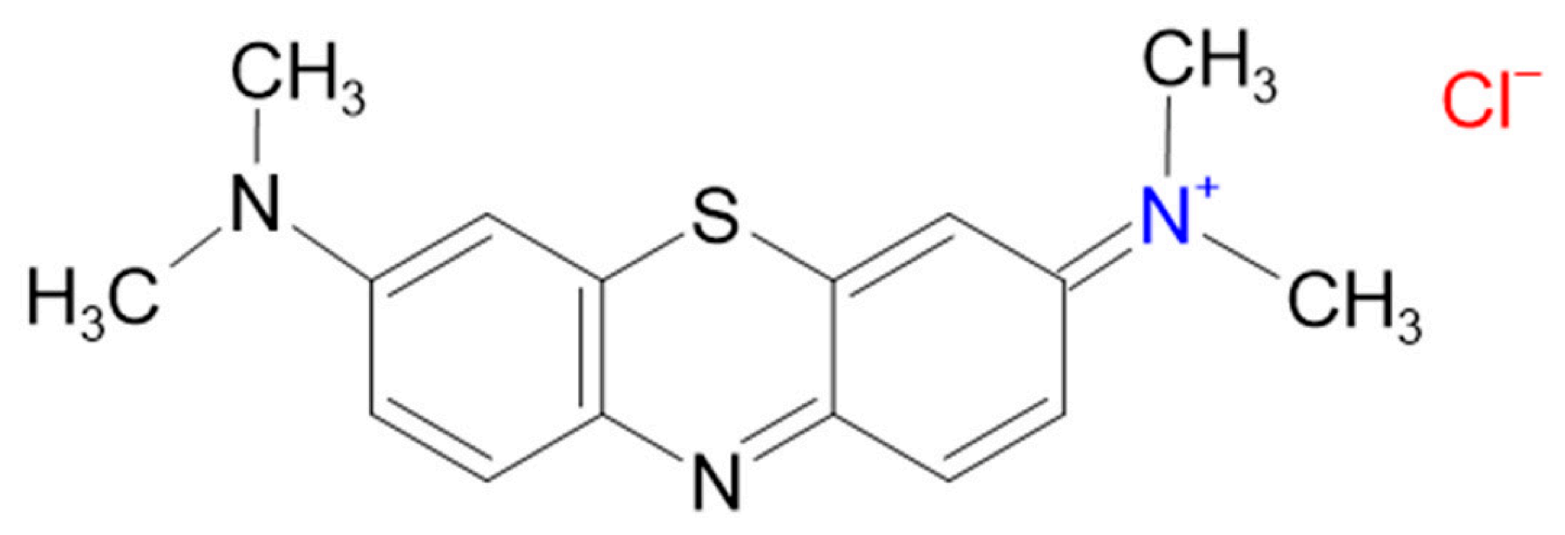
Chemical structure of Methylene Blue dye.

Colored compounds discharged from textile industries pose significant environmental and health hazards due to their carcinogenicity, non-biodegradability, and toxicity. In addition to these concerns, their visible presence in water has drawn considerable attention in recent years [102]. Among the relevant chemical and biological technologies are chemical coagulation and oxidation, photocatalytic oxidation, biological oxidation, and membrane filtration [103,104,105]. Membrane filtration was considered a promising method for dye removal from wastewater [105]. Dyes could be positively charged, like Methylene Blue (MB), or negatively charged, like Rose Bengal (RB). CA-g-IA, containing carboxylic groups, was proposed for the removal of positively charged dye from wastewater. The carboxylic groups deprotonate to give negatively charged sites (-COO^−^), which would enhance the electrostatic interactions with the positive charge present on MB molecules (Figure 11).

The removal process occurred as described in the experimental section.

##### Factors Affecting the Removal of Dyes

To determine the conditions needed to achieve high dye removal, contact time, pH, initial dye concentration and G% were also studied, and the results are presented below.

##### Effect of Contact Time

**Figure 12:**
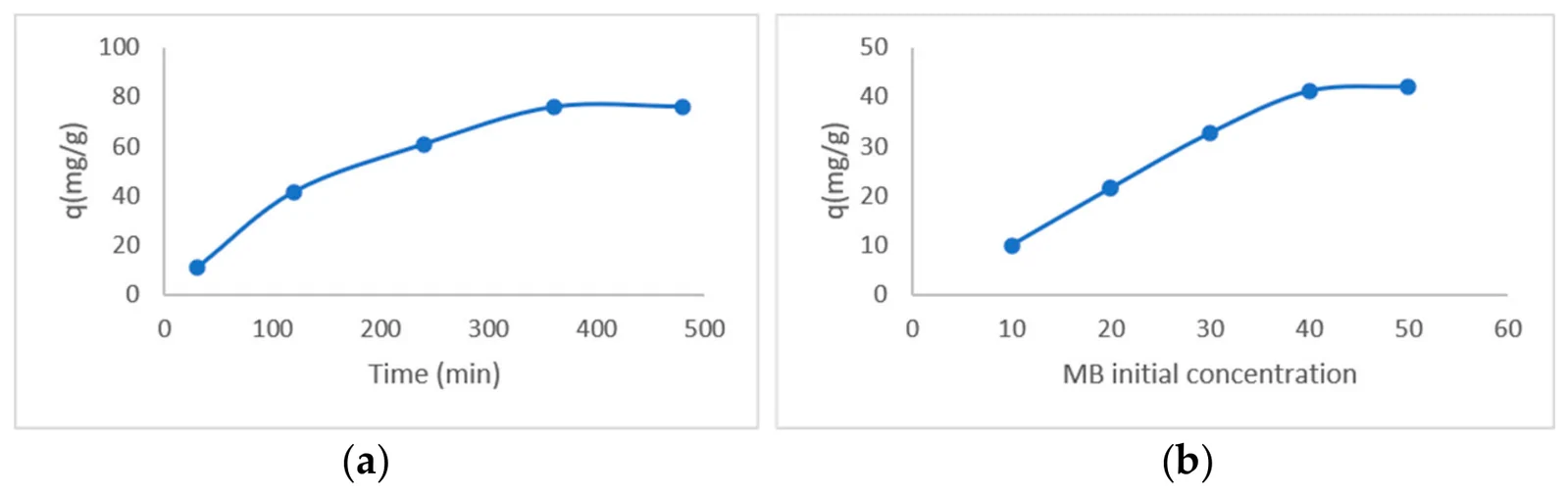
Factors affecting MB dye removal by CA-g-IA membrane. (**a**) Effect of time (**b**) Effect of MB initial concentration.

Figure 12a illustrates the adsorption performance of the CA-g-IA (6.6 G%) membrane for MB removal with an initial concentration of 40 ppm from an aqueous solution at room temperature. The removal capacity increased progressively with contact time and reached equilibrium at approximately 360 min. Beyond this period, no appreciable increase was observed, indicating that the available carboxylate (–COO^−^) active sites in the grafted membrane had become largely occupied by MB molecules [106].

##### Effect of pH

The initial pH of a solution is a crucial factor in determining the capacity of an adsorbent in water treatment, since the pH can change the degree of ionization of the adsorptive molecule as well as the surface characteristics of an adsorbent [107]. The influence of solution pH on MB removal was evaluated at an initial dye concentration of 40 ppm and a contact time of 2 h, with pH adjusted using 0.1 M NaOH or HCl. The removal efficiency varied with solution pH, reaching a maximum of approximately 41% at pH 6, indicating that slightly acidic conditions favored MB removal by the CA-g-IA membrane. This could be attributed to the ionization of the functional groups on the membrane and the electrostatic interaction between the materials and the dye molecules [108]. The alkaline pH values were not investigated as the addition of NaOH would block the (-COOH) groups by transferring them to (-COONa).

##### Initial Dye Concentration

The effect of initial MB concentration on the removal performance was investigated at pH 6 over a contact time of 2 h. Increasing the initial dye concentration enhanced the membrane removal capacity, due to the greater availability of MB molecules for interaction with the active binding sites. The removal capacity increased up to an initial concentration of 40 ppm, beyond which no appreciable improvement was observed, indicating saturation of the available active sites within the grafted polymeric matrix (Figure 12b) [108].

##### Characterization of the Removal of MB by CA-g-IA

**Figure 13:**
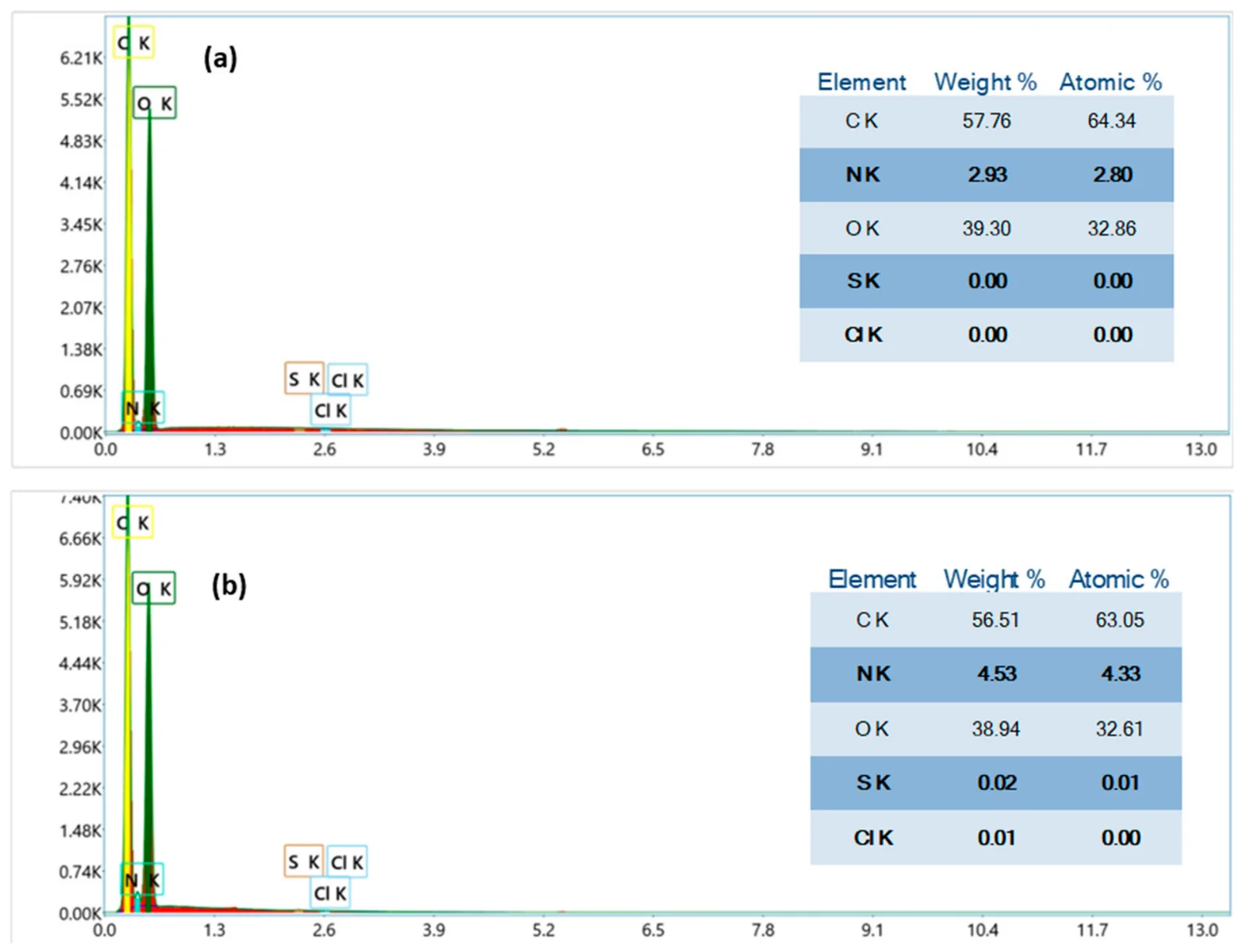
EDS of (**b**) CA-g-IA membrane after MB removal as compared to the ungrafted CA membrane (**a**).

The CA-g-IA (6.6 G%) membrane exhibited a higher MB removal capacity than the pristine CA membrane (Figure 13a). This enhanced performance can be attributed to electrostatic interactions between the cationic MB molecules and the negatively charged carboxylate groups (–COO^−^) introduced through IA grafting (Figure 13b). EDS analysis of the membrane after MB removal further supported dye uptake through the detection of characteristic elements associated with MB, including nitrogen, sulfur, and chlorine.

**Figure 14:**
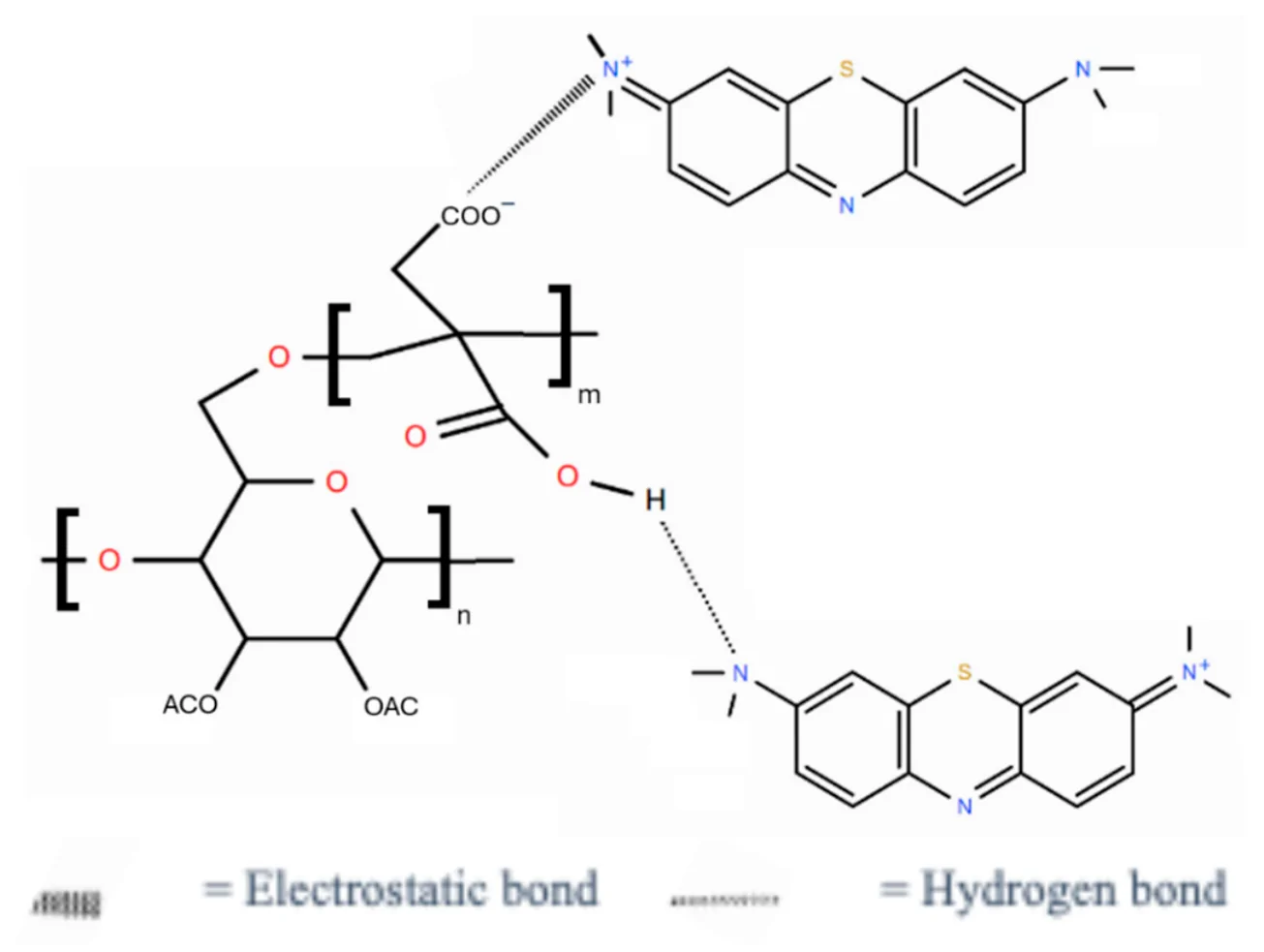
Possible molecular interaction between CA-g-IA membrane and the MB dye.

Table 5 shows experimental pictures showing the higher removal efficiency of CA-g-IA membrane (more intense blue coloration), as compared to the ungrafted CA, under the same removal conditions. Thus, IA enhanced the MB dye adsorption. The binding of dye molecules with the graft membrane occurred through the formation of both electrostatic and hydrogen bonding (Figure 14).

### 3.8. Reusability of the Grafted Membranes

**Figure 15:**
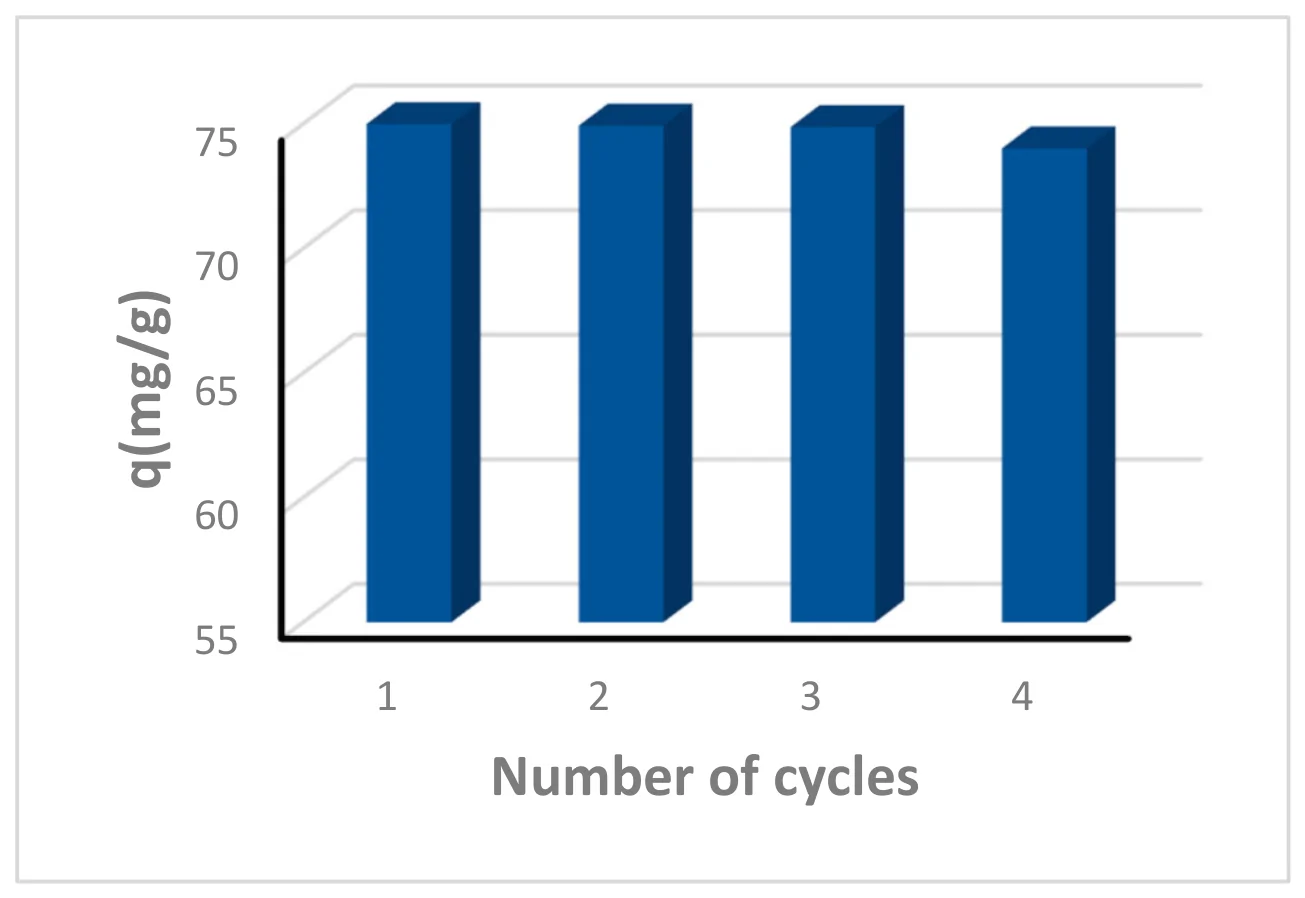
The reusability of CA-g-IA membrane.

The regeneration ability of CA-g-IA membrane was evaluated over four consecutive Cu(II) adsorption–desorption cycles using 0.1 M HNO_3_. After each cycle, the membrane was rinsed, dried, and reintroduced into a new Cu(II) solution under identical conditions. The amount of Cu (II) desorbed was quantified, and the removal capacity (q, mg·g^−1^) was calculated after each cycle based on the residual Cu(II). As shown in Figure 15, the adsorption capacity (q) remained relatively stable throughout the successive regeneration cycles, demonstrating that the membrane retained its Cu(II)-binding performance after repeated use. These results indicate good reusability and regeneration potential of the CA-g-IA membrane under the applied acidic regeneration conditions.

## 4. Conclusions

A novel direct graft copolymerization of bio-based itaconic acid (IA) onto the cellulose acetate (CA) membrane successfully produced a CA-g-IA membrane with additional pollutant-binding active sites, as evidenced by distinct morphological changes on the membrane surface.

The CA-g-IA membrane was characterized using SEM, ^1^H NMR, and UV-Vis spectroscopy to evaluate the morphological and structural changes resulting from the graft copolymerization employed in the study.

The growth of grafted IA branches within the CA polymeric matrix increased the availability of functional binding sites, thereby enhancing interactions with both metal cations and dye molecules.

Graft copolymerization also improved the thermal stability of the CA membrane, as demonstrated by the TGA results. Among the modified membranes, CA-g-IA with 6.6% grafting exhibited the highest thermal stability at elevated temperatures and was more suitable for water treatment applications.

The CA-g-IA (6.6%) membrane also showed the highest Cu(II) adsorption performance, which was attributed to the greater availability of carboxylic functional groups that serve as active binding sites for metal ions.

Increasing the grafting percentage to 13% reduced the membrane performance, attributed to intra- and intermolecular hydrogen bonding among IA moieties within the grafted branches, which decreased the availability of free carboxylic groups for pollutant binding. Cu(II) uptake by the membrane was further supported by XRD and EDS analyses.

In addition to metal ion removal, the CA-g-IA (6.6%) membrane demonstrated effective methylene blue (MB) removal, achieving 41.1% removal at pH 6 and room temperature. The membrane also exhibited excellent regeneration performance, maintaining its removal capacity over four consecutive reuse cycles.

Thus, the novelty of the CA-g-IA membrane lies in the direct grafting of renewable itaconic acid (IA) onto the CA membrane, introducing dual carboxylic functional groups that provide active binding sites for pollutant removal.

## Figures and Tables

**Table 1:** ^1^H NMR spectral data of CA-g-IA, showing five types of protons, referring to the reaction active sites.

CA-g-IA Structure	H-Type	δ (ppm)
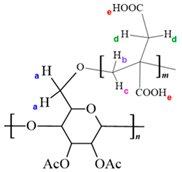	A	1.3
	B	2.4
	C	2.7
	D	1.2
	E	8.6

**Table 2:** TGA of various percentages of CA-g-IA as compared to the parent CA.

Membrane KPS = 0.001997 M, Temp. = 60 °C	T_o_	Weight Loss% at 500 °C
CA	344 °C	91%
CA-g-IA (2.2%)	348 °C	87%
CA-g-IA (6.6%)	351 °C	85%
CA-g-IA (13%)	345 °C	89%

**Table 3:** Mechanical properties of CA-g-IA membranes with various percentages of graft.

Membrane	Tensile Strength (N/mm^2^)	Elongation at Break%
CA	6.90	6.38
CA-g-IA (5%)	7.00	6.59
CA-g-IA (6.6%)	7.2	7.23

**Table 4:** Membrane performance measurement of CA-g-IA membranes with various percentages of graft.

Membrane	Contact Angle of Water Droplet (Degree) (±2)	Water Flux (Lm^−2^h^−1^)
CA	54.9	14.99
Grafted CA (2.2%)	57.2	17.9
Grafted CA (5%)	62.8	27.1
Grafted CA (6.6%)	78.5	28.6

**Table 5:** Pictures of CA and CA-g-IA membranes before and after the removal of MB dye.

CA		CA-g-IA (6.6 G%)	
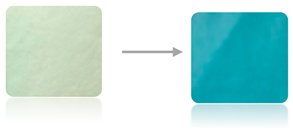		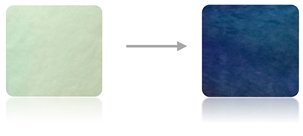	
**Before MB**	**After MB**	**Before MB**	**After MB**

## Data Availability

The raw data supporting the conclusions of this article will be available from the authors on request.
